# Molecular characterization of breast cancer CTCs associated with brain metastasis

**DOI:** 10.1038/s41467-017-00196-1

**Published:** 2017-08-04

**Authors:** Debasish Boral, Monika Vishnoi, Haowen N. Liu, Wei Yin, Marc L. Sprouse, Antonio Scamardo, David S. Hong, Tuan Z. Tan, Jean P. Thiery, Jenny C. Chang, Dario Marchetti

**Affiliations:** 1 Biomarker Research Program Center, Houston Methodist Research Institute, Houston,, 77030 TX USA; 2 Department of Investigational Cancer Therapeutics, The University of Texas MD Anderson Cancer Center, Houston,, 77030 TX USA; 3 Cancer Science Institute of Singapore, National University of Singapore, Singapore, 117599 Singapore; 4 Institute for Academic Medicine, Houston Methodist Hospital, Houston,, 77030 TX USA

## Abstract

The enumeration of EpCAM-positive circulating tumor cells (CTCs) has allowed estimation of overall metastatic burden in breast cancer patients. However, a thorough understanding of CTCs associated with breast cancer brain metastasis (BCBM) is necessary for early identification and evaluation of treatment response to BCBM. Here we report that BCBM CTCs is enriched in a distinct sub-population of cells identifiable by their biomarker expression and mutational content. Deriving from a comprehensive analysis of CTC transcriptomes, we discovered a unique “circulating tumor cell gene signature” that is distinct from primary breast cancer tissues. Further dissection of the circulating tumor cell gene signature identified signaling pathways associated with BCBM CTCs that may have roles in potentiating BCBM. This study proposes CTC biomarkers and signaling pathways implicated in BCBM that may be used either as a screening tool for brain micro-metastasis detection or for making rational treatment decisions and monitoring therapeutic response in patients with BCBM.

## Abstract (web summary)

Characterization of CTCs derived from breast cancer patients with brain metastasis (BCBM) may allow for early diagnosis of brain metastasis and/or help for treatment choice and its efficacy. In this study, the authors identify a unique signature, based on patient-derived CTCs transcriptomes, for BCBM- CTCs that is different from primary tumors.

## Introduction

Multiple studies concur that circulating tumor cells (CTCs)—the “seeds” of fatal metastasis—intravasate into the bloodstream throughout the early stages of cancer promoting the generation of micro-metastatic reservoirs, some of which can progress to macro-metastatic disease^1, 2^. Although it may take years-to-decades for disseminated cancer cells to progress to radiologically detectable metastatic masses^3–5^, by the time it is detected, the metastatic mass usually proliferates at an exponential rate precluding the use of curative treatment options^6–8^. This is particularly true for breast cancer patients with brain metastasis (BCBM), 30% of whom are undiagnosed by current radiological methods and are diagnosed only at autopsy^9^.

We hypothesized that the purported difference in proliferative rates of cancer cells at the opposing ends of the metastatic spectrum should be reflected in the behavior of CTCs that are shed from these sites^10–13^. Accordingly, we posited that characterization of BCBM-associated CTCs may allow for screening/early diagnosis of brain metastasis and help to determine therapies and their effectiveness in specifically targeting BCBM.

In this article, we report the use of a novel workflow for molecular characterization of CTCs isolated directly from breast cancer patient blood. Moreover, we demonstrate that patients with BCBM harbor CTCs with higher expression of proliferation-related biomarkers, and unique signaling mechanisms that may be responsible for brain metastasis.

## Results

### Isolation and characterization of breast cancer CTCs

**Figure 1:**
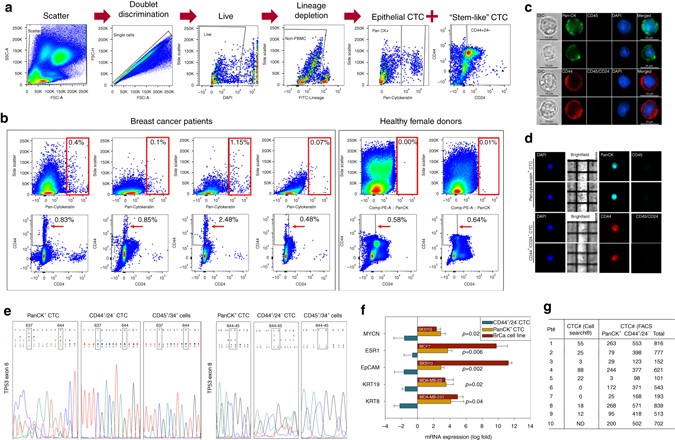
The isolation of CTCs from blood of breast cancer patients using multi-parametric flow cytometry. **a** Flowchart depicting the strategy for isolating breast cancer CTCs by multi-parametric flow cytometry. FITC lineage markers included: CD45—leukocyte, CD73—lymphocyte, CD34—endothelial cell, CD105—macrophage, fibroblast, and CD90—mesenchymal stem cell, hematopoietic stem cell, natural-killer cell. **b** Multi-parametric flow cytometry gating used to isolate epithelial and stem-like breast cancer CTCs (PanCK+, CD44+/CD24− cells) from blood of breast cancer patients. *Red boxes* (*upper panels*) and *arrowheads* (*lower panels*) highlight the absence of these cells applying the same procedures in corresponding blood from healthy female donors. **c** Immuno-cytochemical visualization of patient-derived CTCs by cytoplasmic/cell surface biomarkers. *Scale bar* = 10 μm. **d** Representative images of CTC isolated by the DEPArray^TM^. **e** Chromatogram showing TP53 hotspot mutations in isolated CTCs. *Upper panel*: wild-type sequence; *lower panel*: sequence reads from patient-derived samples. Note the multiple nested peaks of different nucleotides in the boxed cDNA positions. Shown are also patient-derived “normal” CD45+/34+ cells (control) that underwent whole-genome amplification to ensure that altered sequence reads did not arise from the amplification process. **f** mRNA expression pattern of epithelial and neoplastic markers in isolated CTCs. *Error bars* represent s.e.m. (*n* = 3); *p-*value calculated by student’s *t*-test. **g** Summary of CTC number and biomarker parameters in the investigated patient cohort. ND—CTC enumeration not determined on patient#10. Clinical parameters for these patients are provided in Supplementary Table 2

The dynamic nature of epithelial markers expression in breast cancer CTCs along with the substantial presence of de-differentiated cells with higher migratory and tumorigenic properties is well documented^14–16^. Further, because the only FDA-cleared platform for clinical CTC testing—CellSearch®—identifies CTCs based on positivity for the epithelial cell adhesion molecule (EpCAM), cytokeratins (CK), and DAPI, plus absence of the lymphocytic marker CD45^17^, de-differentiated EpCAM—negative or “stem-like” CTCs are excluded^12^. We have addressed this issue by using multi-parametric flow cytometry to capture, isolate, and characterize both epithelial as well as stem-like breast cancer CTC populations^13, 18^. We devised a workflow made of three sequential steps—(i) doublet discrimination and dead cell elimination, (ii) depletion of cells normally present in the peripheral blood using lineage-specific antibodies, and (iii) positive selection of PanCK+ (epithelial) or CD44+/CD24− (stem-like) CTCs (Fig. 1a; Supplementary Fig. 1). We implemented this strategy on a cohort of 10 breast cancer patients and 3 healthy donors. First, we discovered that the patient cohort had a distinct PanCK+ CTC population at an average frequency of 0.486% (of lineage-negative cells) and a mean fluorescence intensity (PanCK-MFI) of 38,757. We also found a second distinct CD44+/CD24− CTC population at an average frequency of 1.381% (of lineage-negative cells) with a CD44-MFI of 16,503. Conversely, we found 0.000% PanCK+ cells and 0.553% CD44+/CD24− cells with a CD44-MFI of 4179, when the same gating strategies were applied on the healthy female donor cohort (Fig. 1b; Supplementary Fig. 1). Thus, the CTC populations isolated were not only unique to breast cancer patients in terms of their increased frequency, but also had a higher cell surface expression of the selected biomarker. These observations are in agreement with prior reports that CTCs are detectable in cancer patients but absent in healthy individuals^19^. Second, immuno-cytochemistry using antibodies against CD45 and CD44 or PanCK showed that isolated CTCs express the appropriate biomarkers used for their selection (Fig. 1c). Third, interrogation of isolated single CTCs using the DEPArray^TM^ platform confirmed the expression of PanCK and CD44 along with the absence of CD45/CD24 biomarkers (Fig. 1d). To further validate the identity of isolated CTCs independent of their cell surface biomarker expression, we performed genomic and mRNA analyses of these cells. Gene exons most commonly mutated in breast cancer patients were PCR-amplified and checked for their mutational status (Supplementary Table 1)^20^. Sanger sequencing showed that isolated CTCs had multiple hotspot mutations in the TP53 gene, whereas “normal” CD45+/CD34+ cells isolated from same patients did not (Fig. 1e). As the healthy female donors had ~0.5% CD44+/CD24− cells, we collected these cells and analyzed exon 6 and exon 8 of the TP53 gene to check whether they harbored any of the breast cancer-associated mutations. Supplementary Fig. 2a shows that these cells were free from mutations in the TP53 gene. Real-time PCR analyses showed that despite possible dilution by the fraction of CD44+/CD24− CTCs with low CD44 expression (as was found in healthy donors), PanCK+ CTCs had 2 to 100-fold higher expression of epithelial markers like KRT8, KRT19, and EpCAM, and 3 to 24-fold higher expression of neoplastic markers like ESR1 and MYCN; whereas CD44+/CD24− CTCs had lower expression of these genes (Fig. 1f). We therefore used this approach, and isolated between 101 and 839 CTCs/8 ml of patient blood, whereas the same subjects yielded between 0 and 88 CTCs/7.5 ml blood when processed in parallel via CellSearch® (Fig. 1g; Supplementary Fig. 2b), implying abilities to capture a larger CTC pool that would have otherwise remained undetected by using only CellSearch®.

### The transcriptomic signature of breast cancer CTCs

**Figure 2:**
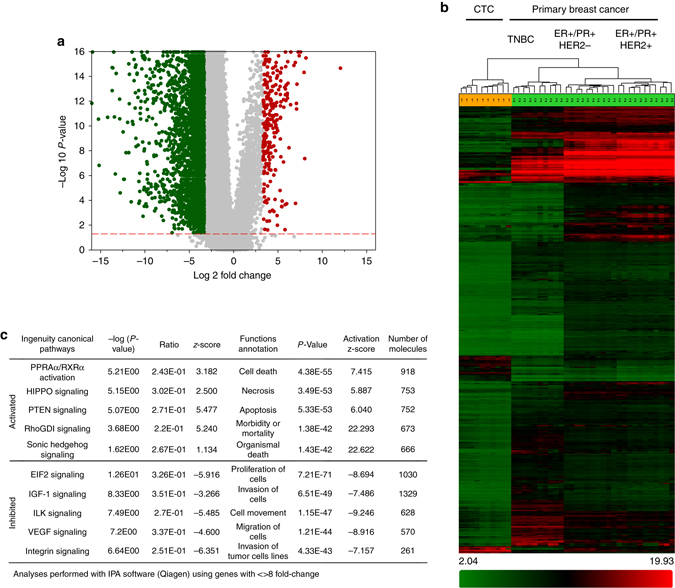
Comparison between CTC and primary breast cancer transcriptomes. **a** Volcano plot showing global gene expression changes in CTCs vs. primary breast cancer (*pBC*). **b** Heat map showing differential expression of 4528 genes in patient-derived CTCs vs. pBC (fold-change <−10 or >10, ANOVA *p*-value <0.05). Note: data from metastatic breast cancer tissue using HTA2.0 array is not publicly available for comparison at present. **c** Significantly altered canonical pathways and cellular functions in patient-derived CTCs compared with pBCs

Next, we performed whole-genome mRNA microarray on CTCs obtained from 10 breast cancer patients with advanced disease (Supplementary Table 2) and compared them to gene expression data curated from 31 primary breast cancer (pBC) samples. These data sets (GSE65505—ER+/PR+/HER2−, GSE67982—ER+/PR+/HER2+, GSE76250—triple negative breast cancer^21–23^) were specifically chosen because they were also derived and analyzed using the same HTA_2.0 platform. As the CTC patient cohort was unmatched with the corresponding pBC molecular subtype, any common signature found in CTCs would be representative of an across-the-board gene signature valid in breast cancer CTCs in general. The two most remarkable observations made from these analyses were: first, 29,758 genes were downregulated in CTCs compared with 1972 genes upregulated (fold-change <−2 or >2, ANOVA *p*-value <0.05), indicative of a generalized low CTC transcriptional activity (Fig. 2a)^14^. Second, none of the CTCs clustered with their corresponding pBC molecular subtype^24^. Conversely, CTCs clustered together as a single group, suggesting they resemble each other more closely than their pBC counterparts^25^ (Fig. 2b; Supplementary Table 2). This notion is further supported by the gene signature unique to CTCs which we found uniformly increased (1972 genes) across the three pBC subtypes. Upon comparison with the Sanger breast cancer cell lines, isolated CTCs clustered most closely with basal-type cell lines (Supplementary Fig. 3a), again indicative of their unique gene signature. Downstream pathway enrichment analyses in CTCs predicted the increased activation of nuclear receptor and pluripotency-related pathways along with decreased protein translational machinery and pro-growth signaling^26, 27^ (Fig. 2c; Supplementary Fig. 3b). These findings were reflected in cellular functions as CTC upregulated genes were involved in cell death, apoptosis, and cell survival with a concomitant decrease in cellular proliferation and invasive properties. Moreover, this trend was further verified by comparing the CTC transcriptome with CD4+ and CD8+ T-cell transcriptomes. Apart from a reduction in pathways related to T-cell function, we detected a similar reduction in the translational machinery and proliferation-related cellular functions (Supplementary Fig. 4a, b; Supplementary Table 3). Deriving from these results and previous investigations^4^, we posited that a sizeable CTC sub-population must exist in a state of reduced metabolic and/or mitotic activity enabling them to overcome stress and survive in circulation^28, 29^.

### The proliferative state of patient-derived CTCs

**Figure 3:**
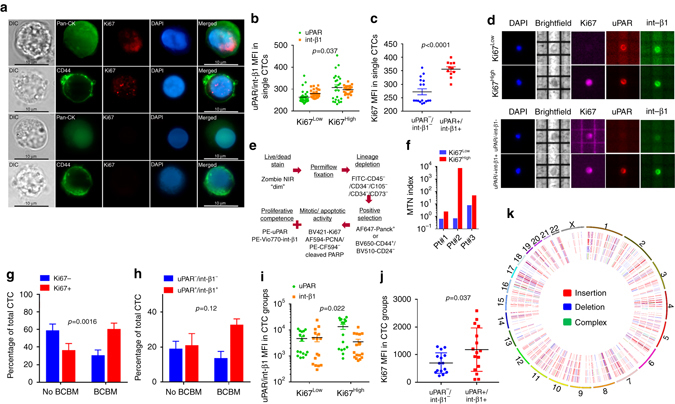
The assessment of proliferation-related biomarkers in breast cancer patient-derived CTCs. **a** Ki67 staining in PanCK+ and CD44+/CD24− CTC subtypes isolated from breast cancer patient blood. *Scale bar* = 10 μm. **b** uPAR and int-β1 expression in single CTCs designated as Ki67^Low^ (*n* = 60) or Ki67^High^ (*n* = 48). *p*-value was calculated by two-way matched ANOVA. Despite a statistically significant overall difference, 20–30% individual CTCs in both groups had similar uPAR and int-β1 expression. **c** Ki67 expression in single CTCs designated as uPAR−/int-β1− (*n* = 17) or uPAR+/int-β1+ (*n* = 11). *p*-value calculated by unpaired *t*-test. Of note, 7 out of 17 uPAR/int-β1 CTCs had higher Ki67 expression than the rest of the group. **d** Demonstration of Ki67, uPAR, and int-β1 staining of single CTCs by DEPArray^TM^. Note that the CTC in the third row, is designated as uPAR−/int-β1, but the Ki67 expression is higher than the Ki67^Low^ CTC shown in the *first row*. **e** Flowchart depicting PBMC processing for concomitant cell surface and nuclear staining for single-step analysis by flow cytometry. PCNA was replaced by cleaved PARP (Asp214) in the last eight samples to accommodate an apoptosis marker in the *nine-color panel*. **f** MTN index of Ki67^Low^ vs. Ki67^High^ CTCs assessed by the Permiflow^TM^ method. **g** Difference in Ki67 status of BCBM vs. no BCBM patient-derived CTCs (*n* = 8). *p-*value calculated by two-way matched ANOVA test. **h** Difference in uPAR/int-β1 expression status in BCBM vs. no BCBM patient-derived CTCs (*n* = 8). *p-*value calculated by two-way matched ANOVA test. **i** uPAR and int-β1 expression in CTC groups designated as Ki67^Low^ or Ki67^High^ (*n* = 32). *p*-value calculated by two-way matched ANOVA. **j** Ki67 expression in CTC groups designated as uPAR−/int-β1− or uPAR+/int-β1+ (*n* = 16). *p*-value calculated by unpaired *t*-test. **k** Circos plot depicting cancer-related gene exon insertions/deletion (in/del) mutations in uPAR+/int-β1+ and uPAR−/int-β1− CTC subsets obtained from BCBM and no BCBM patients. From *outer to inner circle*: uPAR+/int-β1+ and uPAR−/int β1− CTCs derived from BCBM patient; uPAR+/int β1+ and uPAR−/int-β1− CTCs derived from no BCBM patient. 881/884 in/del mutations found in BCBM subsets, whereas 434/211 in/del in no BCBM subsets. *Error bars* represent s.e.m

The biomarker Ki67 is routinely used for assessing the proliferative index of primary breast cancer tissue and is the single most important prognostic factor for breast cancer brain metastasis^30, 31^. Other groups have reported the detection of positive Ki67 staining in CTCs but there has been no systematic study enumerating Ki67+ CTCs by flow cytometry and/or for studying its clinical utility in predicting brain metastasis^32, 33^. As a first step, we stained the isolated CTCs with the Ki67 antibody and detected distinct punctate, nuclear-localized Ki67 staining in both PanCK+ as well as CD44+/CD24− CTC subtypes (Fig. 3a). The observed difference in Ki67 staining intensity was quantified and validated at the single-cell level using the DEPArray^TM^ platform. In addition, we evaluated the cell surface expression of the urokinase plasminogen activator receptor (uPAR) and integrin beta-1 (int-β1), two markers which have been directly implicated in breast cancer dormancy and reactivation both in vitro and in vivo, and compared the expression of Ki67 vis-à-vis uPAR/int-β1 to check whether they corroborated with each other^10, 34, 35^. Single CTC analyses showed that ~50% Ki67^High^ CTCs had higher uPAR expression than Ki67^Low^ CTCs (Fig. 3b). Conversely, ~60% of uPAR−/int-β1− CTCs had low baseline Ki67 expression (Fig. 3c). However, despite the significant concurrence between Ki67 and uPAR/int-β1 expression in CTCs, we detected individual CTCs that showed discordance between Ki67 and uPAR/int-β1 expression (Fig. 3d). These findings suggest that at the single-cell level, neither Ki67 nor uPAR/int-β1 combinatorial expression fully corroborates with each other, thereby warranting further investigation into their complimentary roles in assessing CTC growth arrest^35^. Because both immuno-cytochemical and DEPArray methods of CTC evaluation are semi-quantitative methods involving multiple steps and cannot provide an accurate quantitative estimate of CTCs directly from patient blood, we designed a single-step flow-cytometric method to enumerate Ki67+ and uPAR+/int-β1+ CTCs in patient blood (Fig. 3e). To ascertain whether the Ki67 expression estimated by this flow cytometric method is reflective of CTC proliferative status, we compared the ratio of mitochondrial (mtDNA) to nuclear DNA content in Ki67^High^ vs. Ki67^Low^ CTCs as mtDNA copy number is a high-level indicator of mitochondrial biogenesis thus proliferative potency^36, 37^. Real-time quantitative PCR determination in three individual patient samples showed that Ki67^High^ CTCs had 4 to 10,000-fold higher mitochondrial-to-nuclear DNA (MTN) index when compared with Ki67^Low^ CTCs (Fig. 3f). Having validated the biological difference between Ki67^High^ and Ki67^Low^ CTCs using the MTN index as a surrogate for proliferative status, we analyzed CTCs from 16 breast cancer patients—8 with clinically diagnosed brain metastasis (BCBM—MRI detectable) and 8 without (no BCBM) (Supplementary Table 4). Our results demonstrate that BCBM patients had twice as much Ki67^High^ CTCs (ratio of Ki67^High^:Ki67^Low^~2:1), whereas no BCBM patients had 60% greater Ki67^Low^ CTCs (ratio of Ki67^High^:Ki67^Low^~1:1.6) (Fig. 3g; Supplementary Fig. 5a, b). We also compared the ratio of uPAR−/int-β1− vs. uPAR+/int-β1+ CTCs in these patient samples and found that though there was no significant difference in their expression in no BCBM patients, there was a 2.4-fold increase of uPAR+/int-β1+ CTCs in BCBM patients (Fig. 3g, h; Supplementary Fig. 5c). In addition, we evaluated the apoptotic state of patient-derived CTCs using the cleaved PARP (Asp214) antibody. Out of the 8 patients tested, we did not detect apoptotic CTCs in 4 patients, whereas the other 4 patients had between 1 and 16 apoptotic CTCs (Fig. 3i; Supplementary Fig. 5b). No Ki67− or uPAR−/int-β1− CTCs were positive for cleaved PARP except patient#24 (whose 8 out of 16 apoptotic CTCs were uPAR−/int-1β−). Consistent with DEPArray^TM^, flow cytometric analyses also showed that the majority of Ki67^High^ CTCs had higher expression of uPAR/int-β1 (Fig. 3i). Conversely, the majority of uPAR+/int-β1+ CTCs had significantly higher Ki67 expression (Fig. 3j). Consistent with our single-cell CTC analyses, we found that the Ki67 status of 20–30% CTCs do not corroborate with their uPAR/int-β1 expression and vice versa. These results highlight the complementary roles played by each of these biomarker sets in defining distinct CTC subsets. As proliferating neoplasms are typified by increased genomic instability^38^, we isolated uPAR−/int-β1− and uPAR+/int-β1+ CTC subsets from BCBM vs. no BCBM patient pairs (both ER+/PR+/HER2−) and extracted genomic DNA from these cells. Whole-genome sequencing of these subsets demonstrated a significantly higher incidence of genomic mutations not only in BCBM vs. no BCBM pair, but also in uPAR−/int-β1− and uPAR+/int-β1+ CTC subsets (Fig. 3k). Collectively, these differences in biomarker expression along with CTC mutational content indicated that at least a portion of CTCs in BCBM patients’ blood were unique from the remaining population in regard to their biology and behavior.

### Distinct transcriptomic signature of brain metastatic CTCs

**Figure 4:**
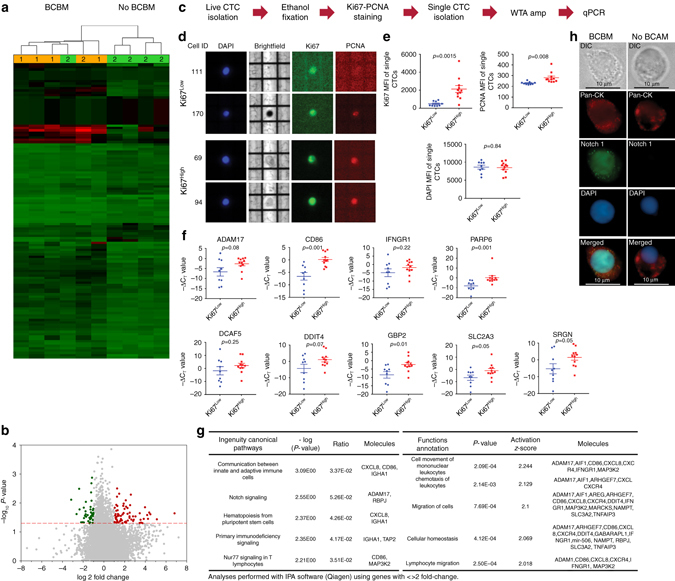
Difference in transcriptomic signatures of BCBM vs. no BCBM CTCs. **a** Heat map showing CTC transcriptomes in BCBM (*n* =  5) vs. no BCBM (*n* = 5). Clustering was performed using genes with fold-change <−2 or >2, ANOVA *p*-value <0.05. Of note, a 126-gene signature was found, of which 73 genes were upregulated and 53 genes were downregulated. Patient# 3 (no BCBM clustering with BCBM) expired before the study results were obtained and could not be followed-up. **b** Volcano plot showing the global gene expression changes in CTC isolated from blood of BCBM vs. no BCBM cases. **c** Workflow for isolating single Ki67^Low^ and Ki67^High^ CTCs. **d** Representative images showing Ki67, PCNA, and DAPI staining in single CTCs. All 20 single CTCs are shown in Supplementary Fig. 6. **e** Mean fluorescence intensity of Ki67, PCNA, and DAPI in isolated single CTCs designated as Ki67^Low^ (*n* = 9) vs. Ki67^High^ (*n* = 11). *p-*values calculated by unpaired *t*-test. Of note, both Ki67 and PCNA staining are significantly higher in Ki67^High^ CTCs, whereas DAPI staining intensity is similar. **f** qPCR analyses showing gene expression levels in single CTCs. *p-*values calculated by unpaired *t*-test. **g** Activated canonical pathways and cellular functions in BCBM CTCs. **h** Representative images showing Notch1 staining in PanCK+ CTCs isolated from blood of BCBM and no BCBM patients. *Scale bar* = 10 μm. *Error bars* represent s.e.m

Next, we aimed to discern CTC signaling pathways associated with breast cancer brain metastasis. It is to be noted that although all 10 patients had advanced metastatic breast cancer, only 5 of them had clinically detectable brain metastases (BCBM) proven by MRI (Supplementary Table 2). Therefore, we postulated that any differential gene expression profile between these two groups should be arising from brain metastatic CTCs. Hence, we used the presence of MRI proven brain metastases to discriminate between these two groups. We discovered that the CTC gene signatures from 5/5 BCBM and 4/5 No BCBM patients clustered in line with their respective clinical groups (Fig. 4a) and presented with a unique 126-gene signature significantly altered between these two groups (Fig. 4b). Because, the 126-gene signature should be arising from CTCs found only in BCBM patients and as our data showed that a significant proportion of CTCs are Ki67+, at least some of the individual Ki67+ CTCs should recapitulate this 126-gene signature. Accordingly, we isolated CTCs by FACS, fixed them with ethanol, followed by staining with Ki67/PCNA/DAPI (Fig. 4c). Using the DEPArray^TM^, we identified and isolated 20 single CTCs, defined as DAPI+ along with variable levels of Ki67 or PCNA staining (Fig. 4d). Isolated single CTCs were classified as Ki67^Low^ (*n* = 9) or Ki67^High^ (*n* = 11) based on Ki67 mean fluorescence intensity (Fig. 4e; Supplementary Fig. 6). Next, we performed qPCR analyses of candidate genes identified within the 126-gene signature. Expression of CD86, PARP6, and GBP2 genes showed remarkable consilience with the BCBM gene signature, i.e., their overall expression was significantly higher in Ki67^High^ CTCs. Expression of ADAM17, DDIT4, SLC2A3, and SRGN genes was also higher in 6–8 out of 11 Ki67^High^ CTCs (Fig. 4f). Further, we found that BCBM CTCs had higher expression of CD44, lower expression of CDH1 along with a generally higher EMT score^39^ (Supplementary Fig. 7a). Subsequent pathway enrichment analyses revealed higher activation of known CTC pathways, i.e., Notch^12^, along with the discovery of novel hematopoietic and immune evasion signaling pathways in CTCs derived from blood of BCBM patients (Fig. 4g; Supplementary Fig. 7b). This was further validated by performing Notch1 immuno-cytochemistry on patient-derived CTCs. A total of 22 CTCs from 3 patients diagnosed with BCBM, and 17 CTCs from 3 patients with no BCBM were analyzed. In the BCBM group, ~72% (16/22) CTCs were positive for Notch staining, whereas 24% (4/17) CTCs were positive for Notch1 staining in the No BCBM group. (Fig. 4h; Supplementary Fig. 7c). As our previous CTC work had linked CD44+/CD24− CTCs to the Notch pathway and BCBM, we isolated CD44+/CD24− CTC populations separately from PanCK+ CTCs and compared the expression levels of Notch target genes in CD44+/CD24− CTCs relative to PanCK+ CTCs. Results showed that ADAM17 levels were non-significantly higher, ITCH (a negative regulator of Notch pathway) were significantly lower, whereas DTX1 (directly regulated by Notch activity) levels were lower in Pt#1 but higher in Pt#2 (Supplementary Fig. 7d). Collectively, these results suggest that upregulation of Notch activity is a feature of BCBM CTCs rather than CD44+/CD24− CTCs. Importantly, cellular functional annotations associated with distant metastases such as cell migration and chemotaxis, which were downregulated in the 10-patient CTC-pBC cohort (Fig. 2c)—were significantly activated in the BCBM vs. no BCBM CTC cohorts (Fig. 4g); along with an increase of pro-inflammatory chemokines (TNF, IL1β, and NF-κB), immunomodulatory networks (CXCL8, CXCR4, CD86), and mitogenic growth factors (PDGF-BB) (Supplementary Table 5). As 4/5 BCBM patients were ER+, whereas 1/5 no BCBM patients were ER+, we wanted to clarify whether estrogen receptor positivity of primary breast cancer had any link with BCBM-associated CTCs. Therefore, we checked the ER/PR/HER2 statuses of patient-derived CTCs in order to evaluate whether they mimicked the hormone receptor statuses of their primary tumors that had been resected earlier. Results showed that patients who were previously diagnosed with ER+/PR+ breast cancer and had presented with metastatic recurrence, harbored between 40 and 60% CTCs that did not correspond to the hormone receptor status of the primary tumor (Supplementary Fig. 8a). Further, we re-analyzed the microarray data by dividing the 10-patient CTC cohort along the estrogen receptor (ER) status of the primary tumor. Results showed that the 51-gene signature associated with the ER+ vs. ER− cohort had no commonality with the 126-gene BCBM-vs.-no BCBM signature (Supplementary Fig. 8b, c). Moreover, pathway analyses under the same conditions (2-fold upregulation or downregulation with ANOVA *p*-value <0.05) did not identify any differentially regulated canonical pathways between the ER+ vs. ER− CTC subgroups.

Cumulatively, these results suggest that growth-arrested CTCs constitute a substantial portion of the CTC population accounting for the anti-proliferative signature of the 10-patient CTC cohort (*n* = 10). However, further segregation of the parent cohort into BCBM (*n* = 5) vs. no BCBM (*n* = 5), highlighted the gene signature of transcriptionally and mitotically active CTCs associated with the BCBM phenotype.

## Discussion

Comparison of the genomic and transcriptomic landscapes between primary and metastatic breast cancer tissues have provided insights into cell clones capable of regenerating cancer in a foreign environment^40–42^. However, a comprehensive understanding of the metastatic cascade is incomplete without analyzing cancer cells in circulation, either on their way to newer metastatic niches or after being shed from established metastases^10, 43^. Clinical studies involving patient-derived tissues have established predictive biomarkers of organ-specific breast cancer metastasis^44, 45^. Cell line and xenograft-based studies have also reported the co-existence of cell clones with disparate phenotypic functions such as cell survival under stress, maintenance of long-term tumor initiating potential as well as rapid proliferation, all of which culminate in the establishment of successful distant metastases^46, 47^. Genomic characterization and targeted gene expression analyses of patient-derived CTCs also point towards the existence of significant heterogeneity within the CTC population that has a role in organ-specific metastases^12, 48^. Building upon these notions, our proof-of-concept study highlights specific biomarkers and transcriptomic characteristics of BCBM-associated CTCs.

The brain microenvironment is unique in its separation from the rest of the systemic circulation forcing any CTC with potential colonizing abilities to brain (but no BCBM onset) or, notably, originating from brain metastasis (BCBM) to undergo additional selection pressure^49, 50^. Therefore, a comparison between BCBM-associated CTCs and CTCs from other metastatic sites, provides an improved understanding of CTC evolution in general, and the dissection of mitotically active/inactive populations of breast cancer brain-homing CTCs in particular. In the present study, we not only confirmed that CTCs associated with clinical BCBM have higher activation of Notch signaling, in line with previous findings from our lab^12^, but also expanded upon these discoveries. We discovered novel inflammatory and immunomodulatory networks that may have vital roles in CTC-driven immune evasion and mitotic reactivation. The relevance of these signaling mechanisms in terms of cancer dormancy and development of brain metastases need to be confirmed by future validation studies.

**Figure 5:**
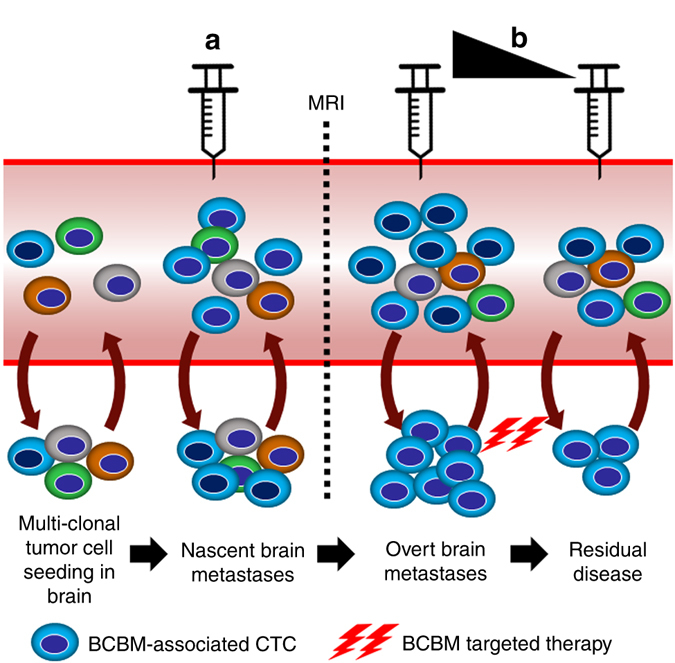
Translational perspectives of employing CTC testing for BCBM patients. Model illustrates clinical implications of BCBM CTC characterization. **a** Enumeration of BCBM CTCs can be used as a screening method for brain micro-metastasis before they become detectable by MRI. **b** Serial estimation of BCBM-associated CTCs can be used as a tool to evaluate responses to therapy for BCBM patients, i.e., the reduction of Notch1+ CTCs and/or Ki67+/uPAR+/int-β1+ CTCs, etc

In conclusion, multiple studies centered upon CTC isolation, enumeration and genomic characterization have established concepts of CTCs as “seeds” of fatal metastases and assessed their relevance as independent prognostic indicators of cancer progression in real-time. This study has focused on the biology of BCBM-associated CTCs and has highlighted signaling mechanisms potentiating CTC growth arrest vs. CTC re-proliferation using BCBM as the clinical discriminator. The clinical implications of our findings are: (a) the application of CTC tests in the clinic as a sensitive screening method for detection of micro-metastatic brain disease, and (b) the use of the newly identified CTC signature for measuring response to therapy for patients with BCBM, i.e., reduction of Ki67+/uPAR+/int-β1+ and/or Notch1+ CTCs (Fig. 5). We foresee that the extension of these studies and/or applications of our findings will allow detection of brain metastasis in its early stage, aid in formulating rational therapies targeted specifically for brain metastases and evaluate the efficacy of these therapies targeted towards brain metastases.

## Methods

### Blood collection and sample preparation

Blood samples were collected from advanced-stage breast cancer patients at MD Anderson Cancer Center according to an IRB-approved protocol. Informed consent was obtained from all human participants. Under strict aseptic conditions, 10–30 ml peripheral blood was obtained (first 5 ml were not used to avoid possible contamination by normal skin cells) and stored in tubes containing CellSave® (Silicon Biosystems/Menarini, Inc.) or EDTA—depending on downstream application. Blood samples from healthy female donors were obtained from the Houston Methodist Blood Donor Center. All blood samples were processed within 24 h of collection. As CTCs are present in the buffy coat fraction, they were enriched by red blood cell (RBC) lysis reagent as previously described^51^.

### Antibodies used for the study

For multi-parametric flow cytometry and DEPArray^TM^, primary antibodies were purchased from the following sources. Anti-human FITC-CD45 (#304054, dilution 1:200), CD34 (#343504, dilution 1:200), CD105 (#323204, dilution 1:200), CD90 (#328108, dilution 1:200), CD73 (#344016, dilution 1:100), APC-Cy7-CD44 (#103028, dilution 1:100) and BV650-CD44 (#103049, dilution 1:100), BV510-CD24 (#311126, dilution 1:100), APC-int-β1 (#303008, dilution 1:100), AF647-Pan-cytokeratin (#628604, dilution 1:100), PE-PCNA (#307908, dilution 1:100), and AF594-PCNA (#307914, dilution 1:100), BV421-Ki67 (#350506, dilution 1:100), PE-uPAR (#336906, dilution 1:100) from Biolegend. PE-Pan-cytokeratin (#5075, dilution 1:100), PE-Estrogen Receptor α (#74244, dilution 1:100), and PE-Progesterone Receptor A/B (#23353, dilution 1:100) from Cell Signaling Technology. BV421-HER2 (#566458, dilution 1:100) and PE-CF594-cleaved PARP (Asp 214) (#564130, dilution 1:100) from BD Biosciences. PE-Vio770—int-β1 (#130-101-276, dilution 1:50) from Miltenyi Biotec.

For immuno-cytochemistry, mouse anti-human CD44 (#960-MSM2-P0, dilution 1:200) and mouse anti-human Pan-cytokeratin antibodies (#MSM2-371-P0, dilution 1:200) were obtained from Neo-Biotechnologies. Rabbit anti-CD44 (ab157107, dilution 1:200) and rabbit anti-Ki67 antibodies (ab66155, dilution 1:200) were obtained from Abcam. Rat anti-Ki67 (#TA801577, dilution 1:800) was purchased from Origene Technologies, and Notch1 antibody (#4380, dilution 1:100) was obtained from Cell Signaling Technology. AF488 and AF594 tagged secondary antibodies and DAPI were purchased from Life Technologies.

### CTC isolation, visualization, and enumeration

For EpCAM-positive CTC capture and enumeration by CellSearch®, 7.5 ml of peripheral blood was collected in CellSave® tubes per CTC assay procedures, and was processed using the Cellsearch® platform (Silicon Biosystems/Menarini, Inc.) following manufacturer’s guidelines. All studies were performed by one of the co-authors (W.Y.) who was blinded to all patient data.

For CTC isolation by multi-parametric flow cytometry, peripheral blood from breast cancer patients was analyzed and sorted using FACSAriaII (BD Biosciences), per strategy outlined in Fig. 1a. Forward scatter area vs. height was used for doublet discrimination and DAPI (DAPI is impermeant to live cells with intact cell membrane) to determine cell viability. FITC was used as “dump” channel and FITC+ cells were eliminated from downstream analyses. For the Permiflow method of CTC analyses (Fig. 3c), Zombie NIR live/dead fixable dye (Biolegend) was used to define viability status prior to fixation. All antibody dilutions were empirically determined using appropriate negative and positive controls. Compensations matrices were constructed using unstained, and single fluorophore stained Versacomp beads (Beckman Coulter, Inc.) and applied before analyses. Data recorded during cell sorting were analyzed by FlowJo_V10 (Ashland, OR, USA).

For immuno-cytochemistry, CTCs were sorted under sterile conditions directly into poly-l-lysine coated glass bottom 96-well plates containing 100 μl culture media, and incubated for 6–12 h—allowing cells to attach. Next, cells were washed with PBS, fixed with 4% PFA, permeabilized with 0.25% Triton-X 100 followed by standard immuno-cytochemistry employing selected antibodies. Cells were then detected using the Zeiss Axio Observer microscope Z1 (Carl Zeiss, Jena, Germany) with enabled ApoTome attachment to allow optical sectioning of fluorescence under ×100 magnification with oil immersion. Images were captured using the Zeiss ZEN 2 software (Carl Zeiss). For visualization as single cells, CTCs were first enriched using flow cytometry, then fixed with 2% paraformaldehyde for 20 min at 25 °C, permeabilized with 0.25% Triton-X 100 for 30 min, and afterwards incubated with appropriate antibodies. Subsequently, cells were washed twice with the SB115 buffer before being loaded into the DEPArray^TM^ cartridge (Silicon BioSystems, Inc.)^35^. Analyses were performed using the custom Fixed_Low_Density program of the DEPArray v3.0 platform. CTCs were detected by the presence of DAPI-stained nuclei and cellular morphology and breast cancer CTC markers. Mean fluorescence intensity of Ki67, uPAR, and int-β1 of single CTCs was obtained and used to evaluate the CTC proliferative status.

### Genomic and mRNA characterization of isolated CTCs

Breast cancer CTC populations isolated by FACS were sorted into a 1.5 ml lo-bind tube (Eppendorf) containing 1 ml sterile PBS and centrifuged in a swing-bucket rotor at 400 rcf for 10 min. Paired FITC-CD45+/CD34+/CD105+/CD90+/CD73+ cells were collected from the same patient in separate tubes to be used as control. Collected cells were subjected to whole-genome amplification using the Repli-G WGA kit (Qiagen). Portions of genes frequently mutated in breast cancer patients were PCR-amplified using the hi-fidelity Q5 polymerase (NEB), treated with Exo-SAP (Affymetrix), gel-purified and subjected to Sanger sequencing at the DNA sequencing and gene vector core facility at Baylor College of Medicine (Houston, TX, USA)^20^. Chromatograms were analyzed using the Chromas software.

For qPCR studies, CTCs were collected in sterile PBS, washed with PBS, and mRNA was amplified using the Repli-G single-cell WTA kit (Qiagen). The cDNA was treated with Exo-SAP (Affymetrix), diluted 1:50 and used for qPCR analyses. For cell lines used as positive controls, mRNA was extracted with the Trizol method, reverse transcribed using the SensiFast kit (Bioline). qPCR was performed using the SensiFast Hi-ROX SYBR mix (Bioline) and run on a 7500 Real-Time PCR system (Applied Biosystems). Supplementary Table 6 lists all the primers used for genomic sequencing and real-time PCR^52^. GAPDH was employed as loading control and fold expression was calculated using ΔΔ*C*_T_ method employing the WTA kit amplified “normal” CD45+/CD34+ cells’ mRNA as control.

### Cell surface and intra-nuclear flow cytometry and mitochondrial DNA assessment

We used the Permiflow method (US patent# US7326577 B2) of cell fixation for concomitant cell surface (e.g., CD45/CD44), cytoplasmic (e.g., PanCK), and intra-nuclear staining of target proteins (e.g., Ki67/PCNA) in patient-derived CTCs^53, 54^. Briefly, isolated patient-derived PBMCs were incubated with the live/dead fixable Zombie NIR dye (Biolegend), washed with PBS, then fixed and permeabilized with the Permiflow solution by incubating samples for 1 h at 42 °C. Cells were then washed with PBS and staining buffer, and subsequently stained with appropriate antibodies for flow cytometric analysis and cell isolation.

The relative number of mitochondrial DNA (mtDNA) to nuclear DNA (nDNA) copies in Ki67^High^ vs. Ki67^Low^ cells was used as a means to verify biological activity and proliferative status^36, 37^. The mitochondrial target was strategically selected within the displacement loop (MTDL), a non-coding region, of the mtGenome because of the rare occurrence of large-scale deletions that are common to other areas, e.g., the major arc^55^. The nuclear target is located within the Beta-2-Microglobulin (B2M) gene and was selected because it is single-copy and has low variability. Supplementary Table 6 lists the primer sequences^56^. Relative quantitative PCR (qPCR) was performed and the mtDNA to nDNA (MTN) index was calculated as MTN index = 2 × 2^ΔCT^, where ΔCT = (CT_B2M_ − CT_MTDL_)^57^.

### RNA microarrays and pathway analyses

CTCs were isolated using a BD FACSAriaII (BD Biosciences) housed inside a BSL-2 facility allowing for collection of CTCs in sterile conditions. The flow cell was cleaned and sterilized before and after running each patient sample. Sorted cells were collected directly into a pre-chilled tube maintained at 4 °C containing RNA lysis buffer and total RNA was collected according to the manufacturer’s protocol (Macherey-Nagel, Inc.). Subsequently, RNA and cDNA amplifications, quality controls and gene expression arrays were performed at the Sequencing and non-Coding RNA Program Core (MD Anderson Cancer Center, Houston, TX, USA) using the HTA 2.0 gene chip (Affymetrix, Inc.). Whole-gene expression data from 31 breast cancer patient samples representing each of the most common molecular subtypes were obtained from three publicly available GEO data sets (GSE65505−ER+/PR+/HER2−, GSE67982−ER+/PR+/HER2+, GSE76250—triple negative breast cancer^21–23^. Whole-gene expression of CD4+ and CD8+ T-cells were obtained from GSE73079 and GSE73081, respectively^58, 59^. Each of these data sets were chosen because they used the HTA_2.0 platform—making the array data compatible for comparison with the CTC transcriptome. Breast cancer CTC samples on Affymetrix HTA-2.0 array were RMA-normalized using Affymetrix Powertool 1.18.0 and annotation was taken from Affymetrix version na36. Epithelial–mesenchymal transition (EMT) scores were computed using a method previously described^39^. Gene expression data from each of these subsets was analyzed using Expression Console and the Transcriptome Analysis Console 3.0.0.466 (Affymetrix). Subsequent pathway enrichment analysis was performed using the ingenuity pathway analysis software (version 01-07; Qiagen, Inc.).

### qPCR analyses of CTC populations and single CTCs

For comparative analysis of CTC populations, CD44+/CD24− and PanCK+ populations from the same patient were collected in two separate tubes and subjected to WTA. Product was treated with Exo-SAP (Affymetrix), diluted 1:50 and subjected to qPCR analysis. Expression levels of Notch pathway-related genes were calculated in CD44+/CD24− CTCs compared with PanCK+ CTCs.

For single CTC analyses, patient-derived PBMCs were subjected to FACS and CTCs were directly collected into a 1.5 ml lo-bind Eppendorf tube containing pre-chilled 80% ethanol with RNasin RNase inhibitor (Promega). CTCs were fixed for 2 h in −20 °C. Afterwards, ethanol was washed out first using sterile PBS, followed by the antibody staining buffer. CTCs were then incubated with Ki67 and PCNA antibodies along with DAPI for 20 min, and washed twice with SB115 buffer and loaded into the DEPArray for single-cell isolation. 20 single CTCs were collected and designated as Ki67^High^ or Ki67^Low^ based upon the mean fluorescent intensity of Ki67 staining. Next, CTCs were washed with PBS and subjected to whole-transcriptome amplification using the REPLI-g WTA single-cell kit (Qiagen). cDNA was treated with Exo-SAP, diluted 1:50 and used for qPCR analysis. *C*_T_ values were normalized to beta-actin (loading control) and −Δ*C*_T_ values of single CTCs were derived as a scatter plot.

### Whole-genome sequencing

Whole-genome amplification was performed using FACS-sorted (CD45−/CD44+/CD24−/EpCAM− and uPAR/int-β1) CTC subsets^35^ and REPLI-g WGA single-cell kit (Qiagen), according to manufacturers’ instructions. Whole-genome DNA library was generated using Illumina Hyper Sample Preparation Kit (KAPA Biosystems, Inc.). Next, generation sequencing at 25× coverage was performed on a HiSeq 2500 Sequencing System (Illumina, Inc.) at the Genomic and RNA Profiling Core facility at Baylor College of Medicine (Houston, TX, USA).

Bioinformatics analyses were performed at the biomedical-informatics core facility at Houston Methodist Research Institute. Briefly, sequence reads were aligned with 1000 genomes reference sequence (v37) with BWA-MEM applied GATK^60^ base quality-score recalibration. Standard hard-filtering parameters or variant quality-score recalibration were done according to GATK Best Practices recommendations^61, 62^. Further, we used Freebayes for variant calling after merging the alignment results for each of the four groups. Lastly, the SnpEff^63^ and GATK were used for annotating the variant calling results. VCFtools^64^ and Samtools^65^ were used for variant filtering. QUAL scores were calculated with a 13-threshold for accuracy (*p*>95%) by −10×log (1−*p*) formula.

### Data availability

The authors declare that all the data supporting the findings of this study are available within the article and its Supplementary Information files and from the corresponding author upon reasonable request. Microarray data is available from the Gene Expression Omnibus as GSE99394. https://www.ncbi.nlm.nih.gov/geo/.

## Electronic supplementary material

Supplementary Information
