# Supplementary material for: Molecular characterization of breast cancer CTCs associated with brain metastasis
Source: Nat Commun. 2017 Aug 4;8:196. doi: 10.1038/s41467-017-00196-1 (PMC5543046; doi:10.1038/s41467-017-00196-1)
Supplement: Supplementary file 1 — Supplementary Information [file 41467_2017_196_MOESM1_ESM.pdf]

File Name: Supplementary Information
Description: Supplementary Figures and Supplementary Tables

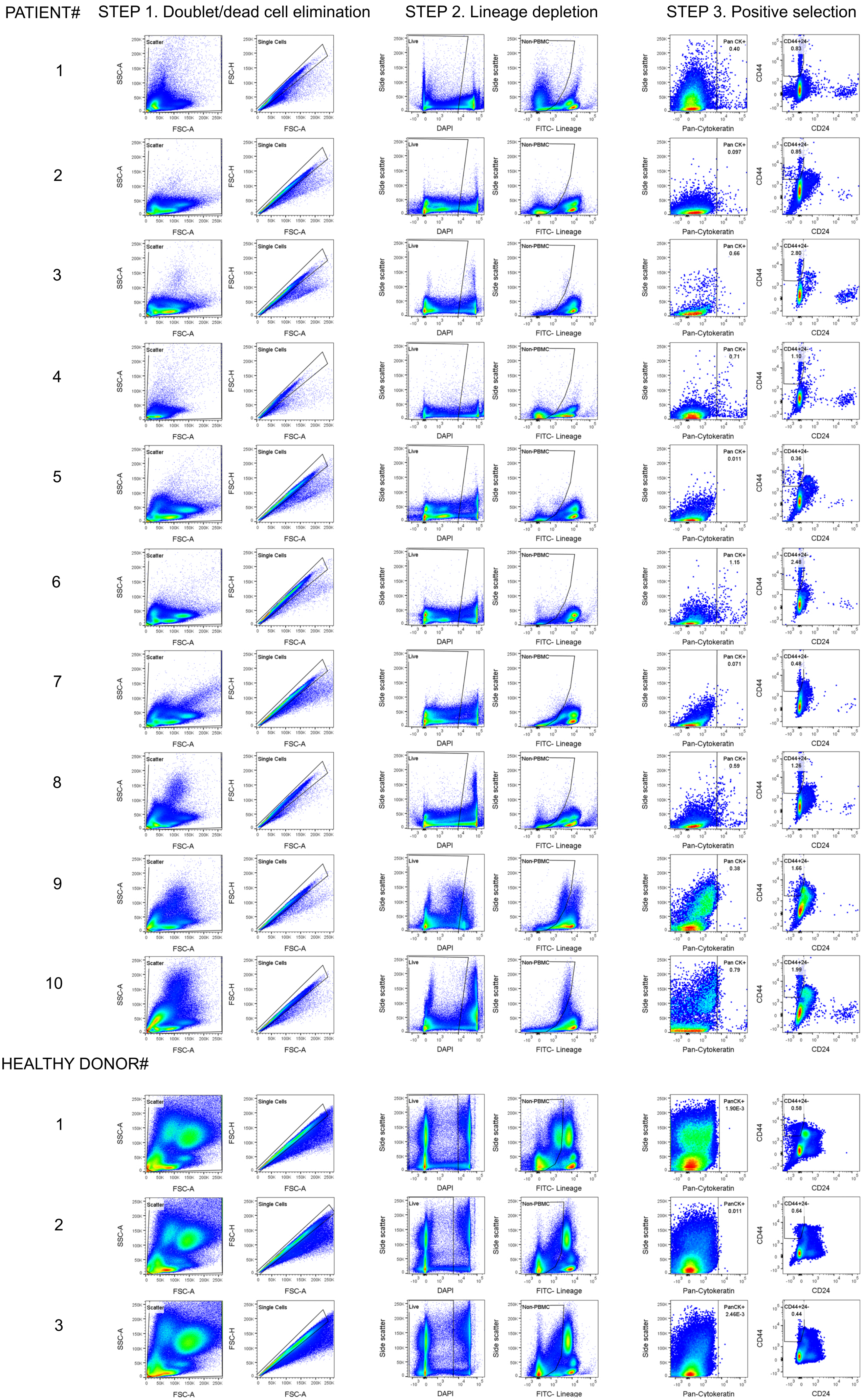
PATIENT#
STEP 1. Doublet/dead cell elimination
STEP 2. Lineage depletion
STEP 3. Positive selection
1
2
3
4
5
6
7
8
9
10
HEALTHY DONOR#
1
2
3
Scatter
Single Cells
Live
Non-PBMC
Pan CK+
CD44+24-
PanCK+
SSC-A
FSC-A
FSC-H
Side scatter
DAPI
FITC- Lineage
Pan-Cytokeratin
CD44
CD24
0.40
0.83
0.097
0.85
0.66
2.80
0.71
1.10
0.011
0.36
1.15
2.48
0.071
0.48
0.59
1.26
0.38
1.66
0.79
1.99
1.90E-3
0.58
0.011
0.64
2.46E-3
0.44

Supplementary Figure 1. The isolation of CTC populations from blood of breast cancer patients and healthy female donors by multi-parametric flow cytometry

A first-step gating strategy was applied to deplete doublets and dead cells (DAPI-negative selection). A second step depleted cell lineages typically found in the peripheral blood allowing for enrichment of atypical cell populations within the peripheral blood mononuclear cells (PBMCs) (CD45-, CD34-, CD90-, CD105-, CD73- cells). A third step gating strategy was then applied for the isolation of PanCK+ (a neoplastic marker) and CD44+/CD24- (a breast cancer stem cell signature) cells from atypical PBMCs isolated from 10 breast cancer patients and 3 healthy female donors (negative controls).

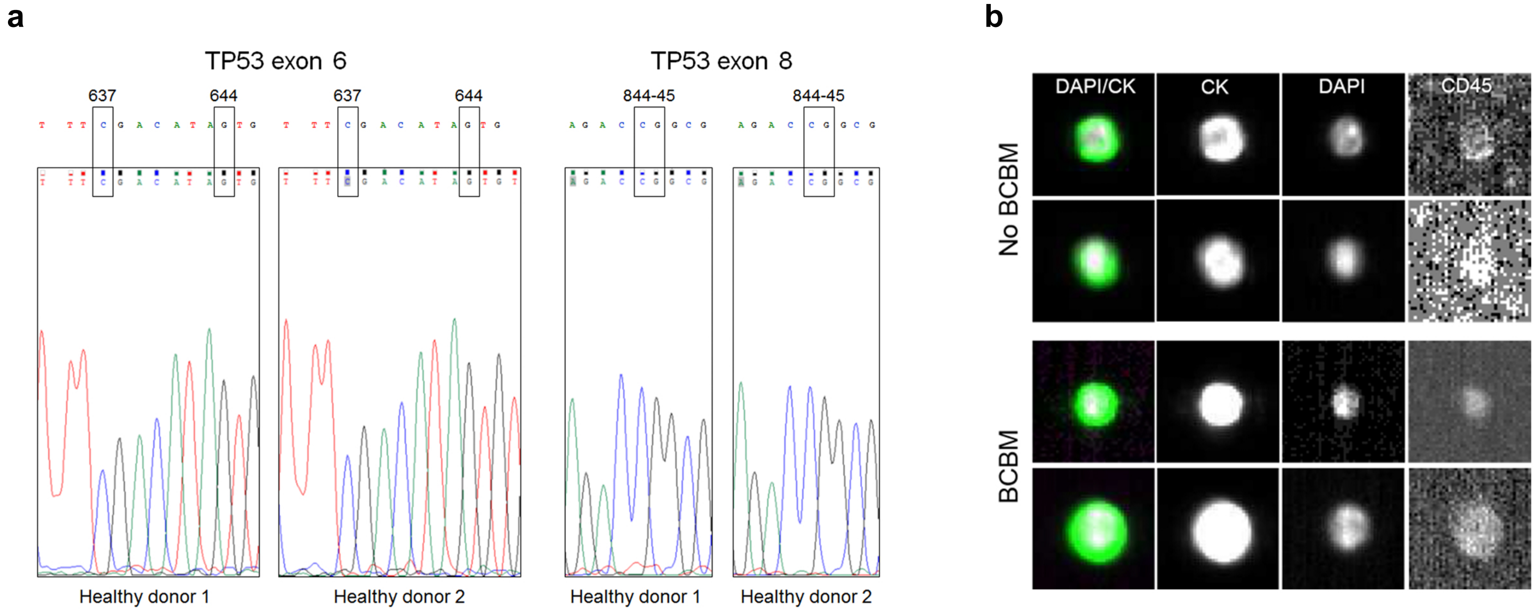

Supplementary Figure 2. Characterization of CD44+/CD24- cells in healthy donors and EpCAM+ CTC enumeration in breast cancer patients

a, Sanger-sequencing of TP53 gene exon 6 and exon 8 in CD44+/CD24- cells isolated from healthy female donors. No overt sequence mutations or heteroplasmic sites were detected. **b,** Representative images of EpCAM+/CK+/DAPI+ but CD45- CTCs captured by CellSearch® from peripheral blood of breast cancer patients diagnosed with (BCBM) or without brain metastasis (No BCBM) are shown.

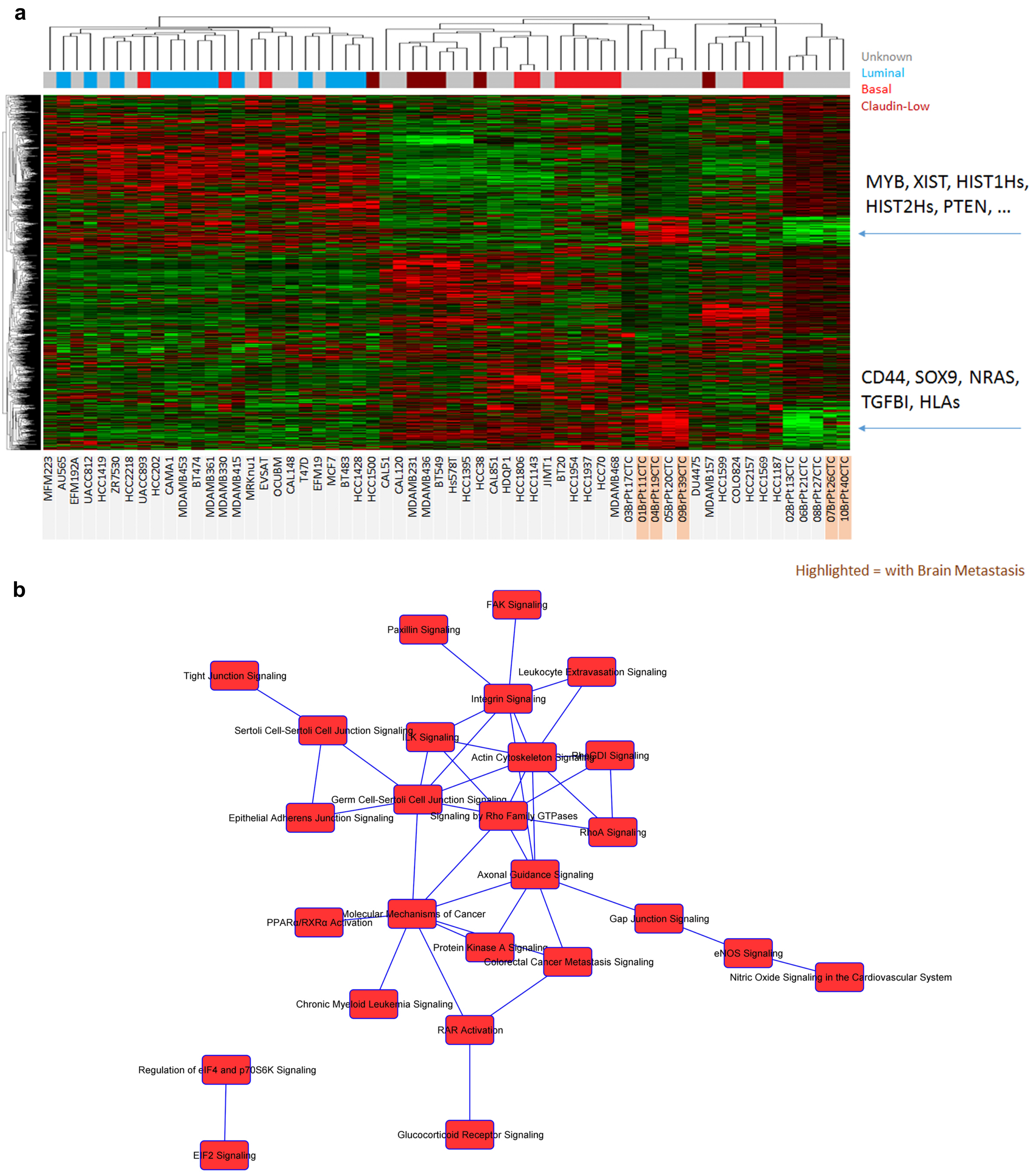

Supplementary Figure 3. Transcriptomic signature of patient-derived CTCs

a, Cluster analyses of isolated CTCs with Sanger breast cancer cell lines showing that CTCs cluster most closely matched with basal subtype cell lines. Key genes associated with this molecular subtype are highlighted. **b,** Network of overlapping canonical pathways significantly altered in isolated CTCs vs pBC tissues. 3-fold changes in gene expression were used for the identification of networks. Top 30 significantly altered pathways with minimum 20 common molecules are shown in organic form. Common pathways derived by analyses using the Ingenuity Pathway Analysis software.

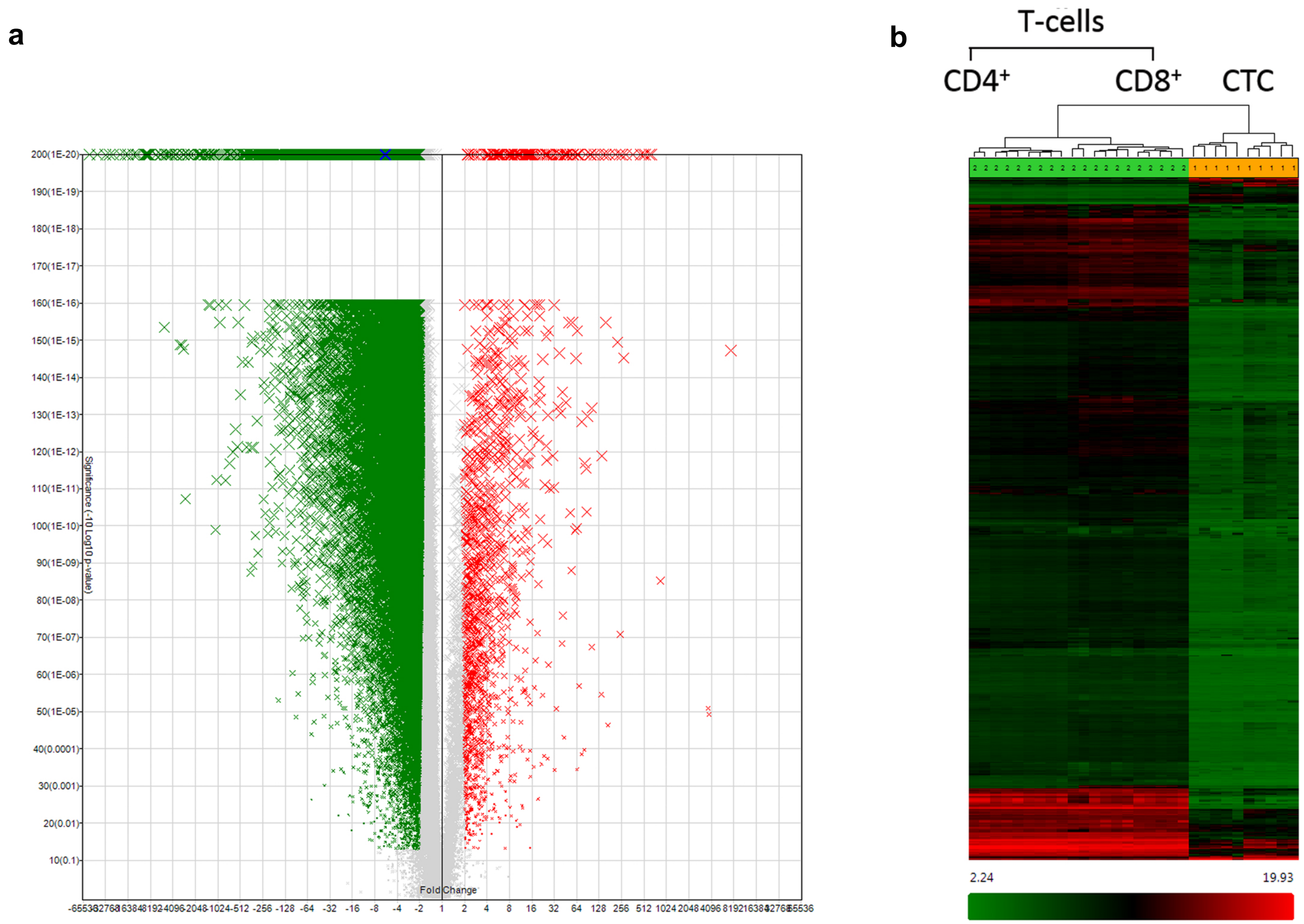

Supplementary Figure 4. Comparative analyses of transcriptomic signatures of CTCs vs CD4+/CD8+ T-cells
a, Volcano plot showing significant gene changes between CTCs vs CD4+/CD8+ T-cells. 1,386 genes were found to be up-regulated while 33,065 genes were down-regulated (fold change < -2 or > 2; ANOVA *p-value* < 0.05). **b,** Heat map showing differential expression of 4,885 genes (fold change <-10 or >10, ANOVA *p-value* <0.05).

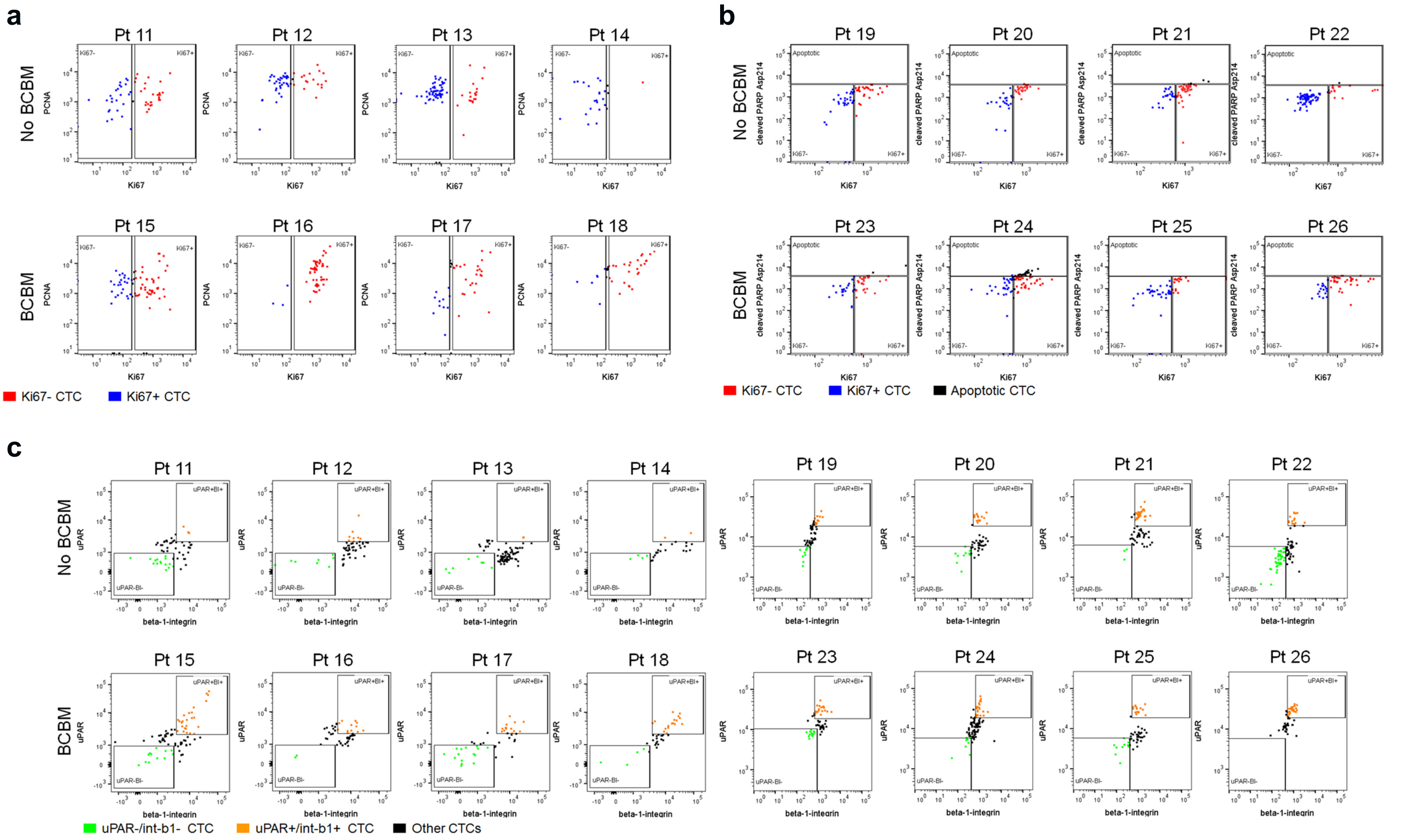

Supplementary Figure 5. Evaluation of proliferation-associated biomarkers in patient-derived CTCs
a, PBMCs from 8 breast cancer patients were analyzed for nuclear expression of Ki67 and PCNA by flow cytometry. Ki67 staining showed a distinct separation between Ki67^{High} vs Ki67^{Low} CTC populations while PCNA staining did not. Hence, all downstream analyses were performed using Ki67 as marker of proliferation. **b,** Assessment of apoptosis and proliferation in 8 breast cancer patient-derived CTCs. Cleaved PARP (Asp214) was used as marker of apoptosis. Gates for apoptosis were set using taxol-treated MDA-MB-231 cells. **c,** Cell surface expression of uPAR and int-β1 in BCBM vs No BCBM patients. Note that a wide range of CTCs (between 10-80%) had mixed expression pattern of uPAR and int-β1 and therefore could not be classified either as uPAR+/int-β1+ or uPAR-/int-β1-.

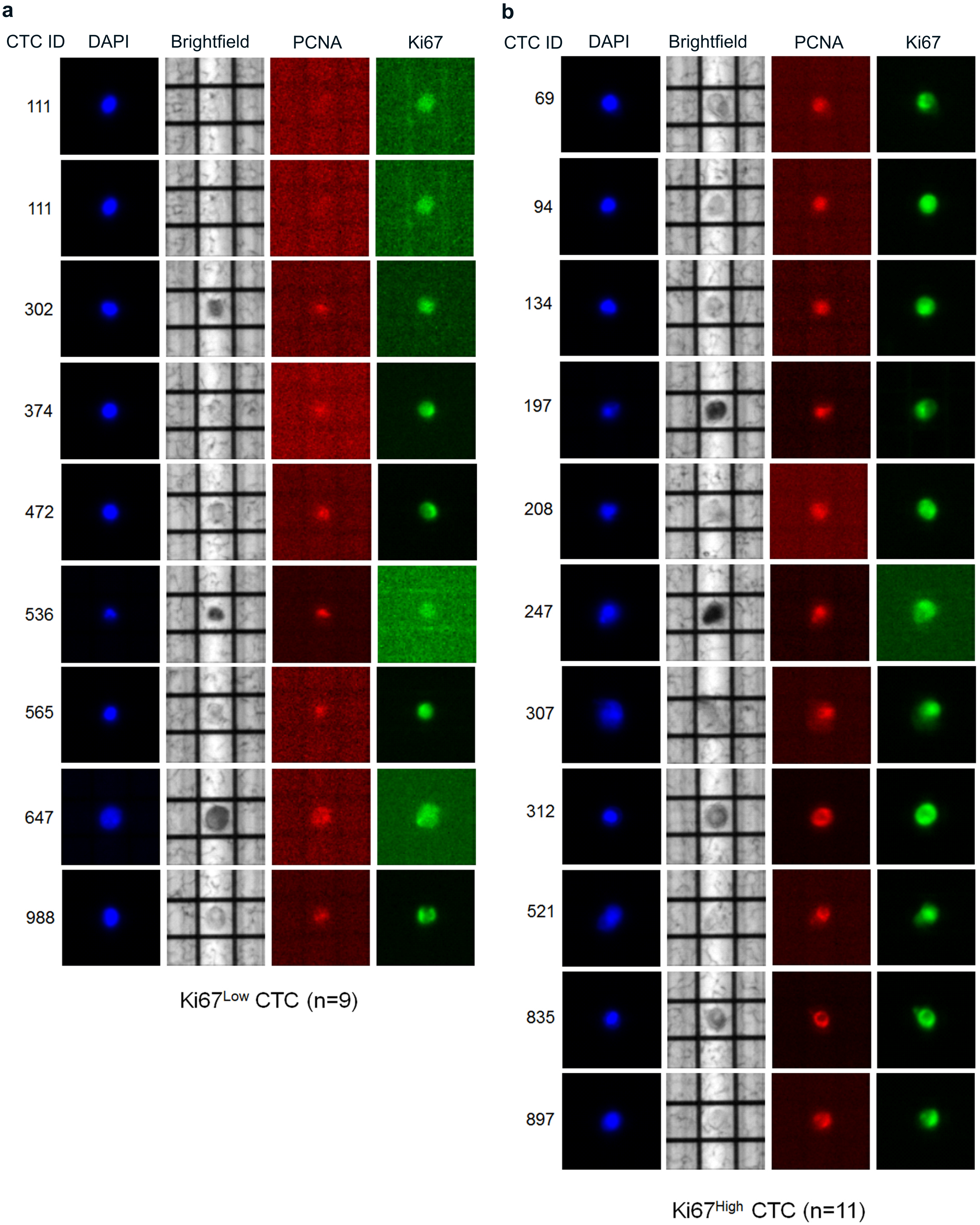

Supplementary Figure 6. Single Ki67^{Low} and Ki67^{High} CTCs isolated from patient blood
CTCs isolated via FACS were fixed with ethanol and stained with Ki67, PCNA and DAPI. Cells were loaded into the DEPArray and single CTCs were classified as **a,** Ki67^{Low} (n=9) or **b,** Ki67^{High} (n=11).

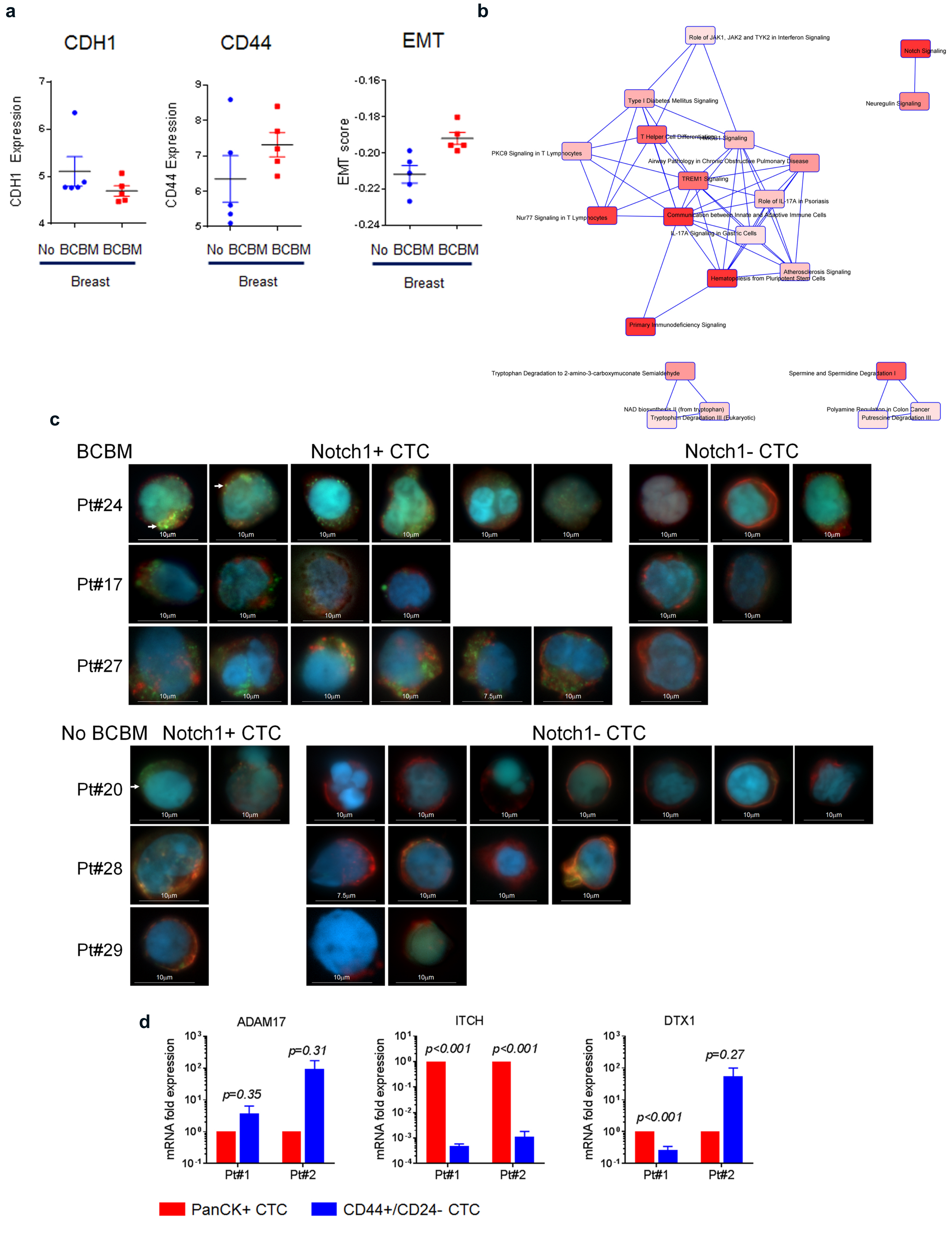
a
CDH1
CD44
EMT
CDH1 Expression
CD44 Expression
EMT score
No BCBM BCBM
Breast
b
Role of JAK1, JAK2 and TYK2 in Interferon Signaling
Notch Signaling
Neuregulin Signaling
Type I Diabetes Mellitus Signaling
T Helper Cell Differentiation
HMGB1 Signaling
PKCθ Signaling in T Lymphocytes
Airway Pathology in Chronic Obstructive Pulmonary Disease
TREM1 Signaling
Role of IL-17A in Psoriasis
Nur77 Signaling in T Lymphocytes
Communication between Innate and Adaptive Immune Cells
IL-17A Signaling in Gastric Cells
Atherosclerosis Signaling
Hematopoiesis from Pluripotent Stem Cells
Primary Immunodeficiency Signaling
Tryptophan Degradation to 2-amino-3-carboxymuconate Semialdehyde
NAD biosynthesis II (from tryptophan)
Tryptophan Degradation III (Eukaryotic)
Spermine and Spermidine Degradation I
Polyamine Regulation in Colon Cancer
Putrescine Degradation III
c
BCBM
Notch1+ CTC
Notch1- CTC
Pt#24
Pt#17
Pt#27
No BCBM
Pt#20
Pt#28
Pt#29
10μm
7.5μm
d
ADAM17
ITCH
DTX1
mRNA fold expression
p=0.35
p=0.31
p<0.001
p=0.27
Pt#1
Pt#2
PanCK+ CTC
CD44+/CD24- CTC

Supplementary Figure 7. Difference between BCBM vs No BCBM CTCs

a, General EMT score, CDH1 and CD44 mRNA expression in BCBM vs No BCBM CTCs. **b,** Network-derived from pathway enrichment analyses of breast cancer CTCs with brain metastases (BCBM) vs without brain metastases (No BCBM). Shown are overlapping canonical pathways significantly activated in BCBM CTCs. **c,** Notch1 immunofluorescent staining in CTCs derived from BCBM vs No BCBM patients. Cytoplasmic red staining: PanCK, cell-surface red staining: CD44, green: Notch1, blue: DAPI stained nucleus. Arrowheads show punctate Notch1 foci in CTCs. A total of 22 CTCs from 3 patients diagnosed with BCBM, and 17 CTCs from 3 patients with No BCBM were analyzed. 72% (16/22) CTCs were positive for Notch1 staining in the BCBM group, whereas 24% (4/17) CTCs were positive for Notch1 staining in the No BCBM group. Scale bar = 10µm or 7.5µm. shown on individual CTCs. **d,** Difference in ADAM17, ITCH and DTX1 gene expression levels in CD44+/24- CTCs as compared to PanCK+ CTCs. *p-values* calculated by unpaired t-test. Error bars represent s.e.m. (n=5).

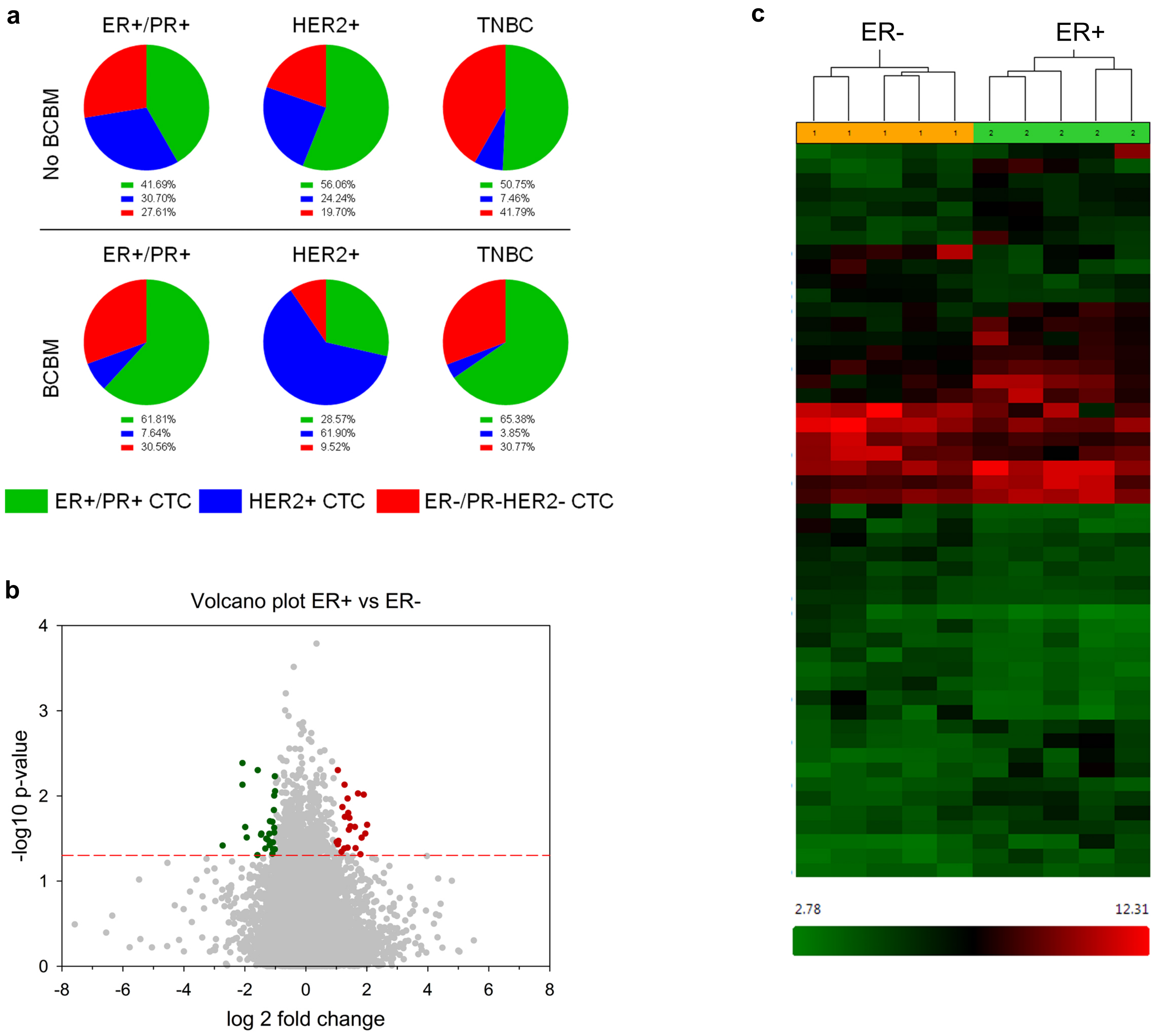

Supplementary Figure 8. Hormone receptor status of CTCs vs primary breast cancer subtypes
a, CTCs from 6 metastatic breast cancer patients were analyzed for ER/PR/HER2 status of CTCs. Results show that patients with prior ER+/PR+ primary tumors have ~40-60% ER+/PR+ CTCs; patients with prior HER2+ primary tumors have ~24-62% HER2+ CTCs while patients with prior TNBC primary tumors have ~30-41% ER-/PR-/HER2- CTCs.
b, Volcano plot showing 51-genes significantly up or down-regulated in ER+ group compared to the ER- group.
c, Heat map showing transcriptomes of CTCs divided on the basis of the ER status of primary tumor. ER+ (n= 5) vs ER- (n= 5). Clustering was performed using genes with fold change < - 2 or > 2 (ANOVA *p-value* < 0.05).

Supplementary Table 1. Gene mutations detected in isolated breast cancer CTCs

CTC Population	Gene	exon	cDNA position	Protein change	**PHR
PanCK$^+$	TP53	6	637C>T	R213*	C (42%)<br>T (24%)
CD44$^+$/24$^-$			638G>C	R213P	G (93%)<br>C (7%)
PanCK$^+$			638G>T	R213L	G (25%)<br>T (40%)
PanCK$^+$			644G>A	S215N	G (19%)<br>A (14%)
PanCK$^+$			644G>T	S215I	G (19%)<br>T (67%)
CD44$^+$/24$^-$		8	844C>A	R282R	C (61%)<br>A (28%)
CD44$^+$/24$^-$			844C>T	R282W	C (61%)<br>T (11%)
PanCK$^+$			845G>C	R282P	G (91%)<br>C (9%)
Pan CK+ or CD44+/24-	CDH1		205T>C	F69V	T (71%)<br>C (29%)
			256A>G	K86E	A (69%)<br>G (31%)
			260G>A	R87K	G (62%)<br>A (38%)
Pan CK+ or CD44+/24-	GATA3		868G>T	L290V	T (58%)<br>G (42%)
			881G>T	M294R	T (59%)<br>G (41%)
Pan CK+ or CD44+/24-	ESR1		829A>T	R277W	A (73%)<br>T (27%)
			833G>T	G278V	G (58%)<br>T (42%)
			847G>T	A283S	G (65%)<br>T (35%)
			870C>T	N290N	C (53%)<br>T (47%)
			951T>C	S317S	T (69%)<br>C (36%)
			956T>C	L319S	T (72%)<br>C (28%)
			975G>C	P325P	C (59%)<br>G (41%)

**Peak height ratios (PHR) were calculated by dividing individual peak heights from the electropherogram by the combined peak heights of each detected nucleotide at the designated position.

Supplementary Table 2: Clinical parameters of breast cancer patients

Pt#	Molecular subtype	Metastatic sites	Brain mets (Y/N)
1	TNBC	Lymph nodes	N
2	ER-/PR-/HER2+	Pleural cavity, skin	N
3	TNBC	Lymph nodes, liver,	N
4	ER+/PR-/HER2-	Lymph nodes, liver, bone, lung, nodes	N
5	TNBC	Lymph nodes, retropectoralis muscle, infraclavicular/supraclavicular nodal basin	N
6	ER+/PR+/HER2-	Lymph nodes, lung, liver, bone, brain	Y
7	ER+/PR-/HER2-	Lymph nodes, lung, brain	Y
8	ER+/PR-/HER2-	Lung, bone, brain	Y
9	ER+/PR+/HER2-	Lymph nodes, lung, liver, bone, brain	Y
10	TNBC	Lymph nodes, lung, brain	Y
11	ER+/PR+/HER2-	Lymph nodes, liver, bone, lung	N
12	ER+/PR+/HER2-	Lymph nodes, bone, lung	N
13	TNBC	Lung, diaphragm	N
14	TNBC	Lymph nodes, lung	N
15	TNBC	Lymph nodes, brain	Y
16	ER+/PR-/HER2-	Lymph nodes, adrenal, lung, pancreas, brain	Y
17	ER-/PR-/HER2+	Lymph nodes, lung, liver, intrathoracic LAD, brain	Y
18	ER+/PR+/HER2-	Lymph nodes, bone, lung, liver, brain	Y
19	TNBC	Lymph nodes, bone, lung, liver	N
20	ER+/PR+/HER2+	Lymph nodes, lung, bone	N

21	TNBC	Lymph nodes	N
22	TNBC	Bone, liver, lung	N
23	ER-/PR-/HER2+	Lymph nodes, lung, bone, brain	Y
24	ER+/PR+/HER2+	Lymph nodes, lung, bone, brain	Y
25	ER+/PR low+/HER2+/-	Lymph nodes, bone, liver, brain	Y
26	ER+/PR+/HER2+/-	Lymph nodes, lung, bone, liver, brain	Y

Supplementary Table 3. Pathways and cellular functions significantly altered in CTCs vs T-cells

	Ingenuity Canonical Pathways	-log(p-value)	Ratio	z-score	Functions Annotation	p-Value	Activation z-score	# of molecules
Activated	PI3K Signaling in B Lymphocytes	5.76E00	1.89E-01	4.796	cell death	8.87E-29	4.167	488
	PPARα/RXRα Activation	5.02E00	1.58E-01	3.411	necrosis	3.68E-25	3.809	392
	Apoptosis Signaling	4.9E00	2.02E-01	1.886	adenocarcinoma	7.08E-22	2.000	1006
	Cytotoxic T Lymphocyte-mediated Apoptosis of Target Cells	4.68E00	3.12E-01	0.816	apoptosis	8.69E-22	4.495	380
	PTEN Signaling	3.71E00	1.61E-01	4.243	cell death of tumor cell lines	4.90E-19	2.438	243
Inhibited	EIF2 Signaling	2.03E01	2.88E-01	-5.292	Viral Infection	1.16E-31	-9.075	288
	iCOS-iCOSL Signaling in T Helper Cells	1.45E01	3.15E-01	-5.292	proliferation of lymphocytes	4.72E-31	-3.706	167
	CD28 Signaling in T Helper Cells	1.33E01	2.88E-01	-5.014	proliferation of cells	6.59E-31	-8.073	529
	Regulation of eIF4 and p70S6K Signaling	1.25E01	2.53E-01	-3.051	cell proliferation of T lymphocytes	6.79E-31	-2.802	145
	mTOR Signaling	9.09E00	1.98E-01	-3.578	proliferation of mononuclear leukocytes	8.13E-31	-3.733	168

Analyses performed with using genes with <>16 fold-change.

Supplementary Table 4. Biomarker expression in primary breast cancer vs CTC

Pt#		Primary tumor	CTC					
		Ki67 status	Total	Ki67^{+}	Ki67^{-}	uPAR^{+}/ int-β1^{+}	uPAR^{-}/ int-β1^{-}	Cleaved PARP+
No BCBM	11	Positive	55	28	26	3	15	ND
	12	ND	53	17	35	8	8	ND
	13	Positive	87	20	65	2	8	ND
	14	Positive	25	1	21	2	4	ND
	19	Positive	67	29	32	16	12	0
	20	Positive	53	30	22	26	11	0
	21	Positive	74	49	22	37	3	3
	22	Positive	91	15	74	14	39	1
BCBM	15	Indeterminate	80	44	25	26	12	ND
	16	Positive	52	49	3	12	2	ND
	17	ND	45	24	11	14	15	ND
	18	ND	39	26	7	17	4	ND
	23	Positive	58	35	21	19	13	2
	24	ND	100	60	34	23	8	16
	25	Positive	57	15	37	16	10	0
	26	Positive	55	38	17	27	0	0

ND: Not determined

Supplementary Table 5. Predicted upstream regulators of BCBM CTCs

Upstream Regulator	Exp Log Ratio	Activation z-score	p-value of overlap	Target molecules in dataset
TNF	0.510	3.064	8.49E-04	ADAM17,CD86,CXCL8,CXCR4,GBP2,IFNGR1,KYNU,NAMPT,SAT1,TNFAIP3
IL1B	0.150	3.052	3.80E-07	AIF1,CD86,CXCL8,CXCR4,DDIT4,GBP2,IFNGR1,NAMPT,SRGN,TAP2,TNFAIP3
OSM	-0.190	2.715	5.67E-06	ADAM17,CLIP1,CXCL8,GBP2,IFNGR1,MARCKS,NAMPT,TAP2
IFNG	-0.030	2.635	5.26E-04	AIF1,CD86,CXCL8,CXCR4,GBP2,IFNGR1,KYNU,NAMPT,TAP2
NFkB (complex)		2.438	1.53E-06	CD86,CXCL8,CXCR4,GBP2,KYNU,NAMPT,RBPJ,TAP2,TNFAIP3
PDGF BB		2.428	2.15E-06	AREG,CXCL8,GBP2,NAMPT,SAT1,SLC2A3,TNFAIP3
IL4	-0.090	2.418	3.34E-03	CD86,CLIP1,CXCL8,CXCR4,GBP2,TAP2
CD40LG	-0.050	2.382	3.37E-08	ADAM17,CD86,CLIP1,CXCL8,CXCR4,MARCKS,NAMPT,TAP2,TNFAIP3
IL1		2.344	3.78E-05	ADAM17,AREG,CXCL8,KYNU,SLC2A3,TNFAIP3
IL6	-0.180	2.282	1.95E-03	AREG,CD86,CXCL8,CXCR4,GBP2,NAMPT

Supplementary Table 6. List of primers used for the study

Primers for genomic DNA amplification

Gene	exon	Forward primer (5' - 3')	Reverse primer (5' - 3')	Amplicon (bp)	Tm	% GC content
TP53	6	GCCTCTGATTCCTCACTGAT	TTAACCCCTCCTCCCAGAGA	182	65	52
	8	AGTGGTAATCTACTGGGACGG	ACCTCGCTTAGTGCTCCCTG	141	67	59
CDH1	2	GTCTCCTCTTGGCTCTGC	GCGGCCTCTCTCCAGGT	102	66	70
	3	GAATTTTGAAGATTGCACCGG	CCCACTGTATTCAGCGTGAC	195	63	48
GATA3	4	TGAACTGTGGGGCAACCTCG	CAGCCTTCGCTTGGGCTTAAT	131	69	60
	6	ATTAACAGACCCCTGACTATG	CCAGTGAGTCATGCACTTTTT	100	62	42
ESR1	4	ATGTTGAAACACAAGCGCCA	GTCTGCCAGGTTGGTCAGTAA	264	67	55

Primers for real time PCR

Gene	Forward primer (5' - 3')	Reverse primer (5' - 3')	Amplicon (bp)	Primer bank ID
MYCN	TGATCCTCAAACGATGCCTTC	GGACGCCTCGCTCTTTATCT	113	62750358c2
ESR1	GGGAAGTATGGCTATGGAATCTG	TGGCTGGACACATATAGTCGTT	158	170295801c2
EpCAM	ATGGACCTGACAGTAAATGGG	GCCTTCTCATACTTTGGCATTC	209	
KRT8	CAGAAGTCCTACAAGGTGTCCA	CTCTGGTTGACCGTAACTGCG	194	372466576c1
KRT19	ACCAAGTTTGAGACGGAACAG	CCCTCAGCGTACTGATTTCCT	181	131412244c2
GAPDH	AAGGTGAAGGTCGGAGTCAAC	GGGGTCATTGATGGCAACAATA	102	83641890b1
MTDL	CTAAATAGCCCACACGTTCCC	AGAGCTCCCGTGAGTGGTTA	83	
B2M	GCTGGGTAGCTCTAAACAATGTA TTCA	CCATGTACTAACAAATGTCTAAA ATGGT	95	
ADAM17	GTGGATGGTAAAAACGAAAGCG	GGCTAGAACCCTAGAGTCAGG	93	73747888c1
CD86	TGTACGACGTTTCCATCAGC	GGTGAAGATAAAAGCCGCGT	106	
IFNGR1	TCTTTGGGTCAGAGTTAAAGCCA	TTCCATCTCGGCATACAGCAA	86	167466162c1

PARP6	AGTTCTGGAATGATGACGACTCG	GTGGGTGTCGATACAGGTCAG	99	94536837c1
DCAF5	CCACTTCGGCTGTGTCAATG	CATGTGCCATAGCAGAACCC	97	195539365c1
DDIT4	TGAGGATGAACACTTGTGTGC	CCAACTGGCTAGGCATCAGC	110	56676369c1
GBP2	CATCCGAAAGTTCTTCCCCAA	CTCTAGGTGAGCAAGGTACTTCT	79	38327557c3
SLC2A3	GCTGGGCATCGTTGTTGGA	GCACTTTGTAGGATAGCAGGAAG	123	221136810c1
SRGN	GGACTACTCTGGATCAGGCTT	CAAGAGACCTAAGGTTGTCATGG	137	45935370c3
DTX1	GACGGCCTACGATATGGACAT	CCTAGCGATGAGAGGTCGAG	84	41352717c2
ITCH	TGATGATGGCTCCAGATCCAA	GACTCTCCTATTTTCACCAGCTC	94	380420336c1
ACTB	TCCTCTCCCAAGTCCACACAGG	GGGCACGAAGGCTCATCATTC	131	
